# A Cross-Sectional Study of Acute Coronary Syndrome and Thyroid Profile: Dissecting the Relationship to Improve Patient Care

**DOI:** 10.7759/cureus.55793

**Published:** 2024-03-08

**Authors:** Ritesh Kumar, Rashmi Sinha, Gagan Gunjan, Sanjay K Singh

**Affiliations:** 1 Department of General Medicine, Mahatma Gandhi Memorial Medical College and Hospital, Ranchi, IND; 2 Department of General Medicine, Rajendra Institute of Medical Sciences, Ranchi, IND

**Keywords:** hyperthyroidism, hypothyroidism, euthyroid sick syndrome, thyroid hormone, acute coronary syndrome

## Abstract

Introduction: Thyroid-releasing hormones are pivotal in regulating cardiovascular (CVS) function and maintaining its hemodynamics and homeostasis. Even a minor alteration in thyroid function has an enormous implication on CVS morbidity and mortality. Moreover, hypothyroidism was found to be a potential menace for coronary artery disease (CAD). The objective of this study was to determine the role of thyroid-releasing hormones in patients suffering from acute coronary syndrome (ACS).

Methodology: Among a cohort of 100 patients suffering with ACS, a complete history and clinical information followed by physical examination and electrocardiography were recorded. Blood samples were also collected to record the blood sugar levels i.e., fasting blood sugar (FBS), postprandial blood sugar (PPBS), and thyroid profile, including free thyroid stimulating hormone (TSH), free thyroxine (fT4), free triiodothyronine (fT3), and reverse triiodothyronine (rT3). The data was analyzed using SPSS version 26 software (IBM Corp., Armonk, NY, USA).

Result: The study identified alterations in the thyroid hormone levels in 27% of patients suffering from ACS. The prevalence of euthyroid sick syndrome was found to be 59.3%, while subclinical hypothyroidism and subclinical hyperthyroidism were reported among 18.5% and 14.8% of patients respectively. There was no significant difference found between males and females. The study illustrated a greater occurrence of aberrant thyroid hormone profiles among those aged 40-60 years. The ST-elevated myocardial infarction (STEMI) group had a statistically significant higher prevalence of an aberrant thyroid hormone profile compared to the non-ST-elevated myocardial infarction (NSTEMI) and unstable angina (UA) groups (p=0.02). A total of nine patients died with ACS and all of those had statistically significant low fT3 and TSH values while higher rT3 values (p<0.05).

Conclusion: An atypical thyroid status has been found to elevate the likelihood of developing CAD and experiencing CVS mortality. This condition can impact ventricular function and serum cholesterol levels as well as heart rate and rhythm. Therefore, understanding this relationship could potentially lead to improved treatment strategies for individuals with ACS which will further prevent major CVS complications.

## Introduction

Ischemic heart disease (IHD) and its most dangerous manifestation, acute coronary syndrome (ACS), are the most frequent causes of morbidity and mortality worldwide [1]. ACS is a medical condition that includes any group of clinical symptoms that are compatible with acute myocardial infarction (MI). It is further grouped into ST-elevated myocardial infarction (STEMI), non-ST-elevated myocardial infarction (NSTEMI), and unstable angina (UA) [2]. Thyroid releasing hormone is pivotal in regulating cardiovascular (CVS) function and maintaining its hemodynamic, and homeostasis [3]. An inadequate amount of thyroid hormone in the body leads to the development of hyperlipidemia and ventricular arrhythmias. Conversely, an excessive amount of thyroid hormone results in the occurrence of atrial arrhythmias. Both of these conditions lead to the development of comorbidities such as hypertension and heart failure. Considering this, the American Heart Association's (AHA) recommendation is to assess thyroid function in all patients suffering from heart failure [4].

Multiple investigations have documented the presence of an abnormal thyroid profile among patients suffering from ACS [5-7]. "Euthyroid sick syndrome" stands out as the most prevalent condition in patients grappling with ACS. This condition is characterized by an alteration in the typical feedback regulation mechanism of thyroid homeostasis and presents with reduced levels of serum triiodothyronine (T3) and/or free triiodothyronine (fT3), elevated levels of serum reverse triiodothyronine (rT3), and concurrently normal levels of blood thyroid stimulating hormone (TSH), thyroxine (T4), and free thyroxine (fT4) [5,8,9]. Notably, this condition has been reported in many cases of severe chronic heart failure [10,11] and acute MI [12,13], and as a rapidly arising condition after open-heart surgery [8]. Moreover, hypothyroidism was found to be a potential menace for coronary artery disease (CAD) [14]. Previously, it was believed that CVS disease predominantly impacted nations with higher income levels. Nonetheless, the current scenario substantiates the fact that CVS disease is alarmingly increasing among low- and middle-income countries, including India [15]. Fortunately, cardiac irregularities typically exhibit a potential for reversal on the administration of medication targeting the underlying thyroid disease [3]. Thus, it is important to study the prevalence of thyroid dysfunction and its impact on patients' health outcomes among individuals suffering from ACS.

However, it is worth noting that despite the wealth of research on this topic, there still exists a notable gap in the available data concerning the impact of thyroid hormone abnormalities on aggravating ACS in hospitalized patients. Consequently, this study was conducted with the aim of assessing the profile of thyroid hormones and their correlation with ACS patients.

## Materials and methods

A cross-sectional study was conducted in the Department of Medicine, Rajendra Institute of Medical Sciences (RIMS), Ranchi, India for a duration of 18 months, i.e., April 2020 to September 2021. Purposive sampling was done, and a cohort of 100 consecutive instances of ACS participated in the study.

Before the study began, the Institutional Ethics Committee (IEC number 142 IEC RIMS, Ranchi) granted ethical approval, and written informed consent was obtained from all the participants. The inclusion criteria for the study were any adult over 18 years of age granting informed consent and was diagnosed with ACS or patients with a history of chest pain showing changes in ECG and cardiac biomarkers for MI. However, patients with thyroid disorders who were undergoing treatment or had other medical conditions such as chronic renal failure, neoplasia, cirrhosis of the liver, chronic obstructive lung diseases, any active infective conditions, and individuals who were taking steroids, lithium, amiodarone, or had received iodinated contrast agents within the previous two weeks were excluded from the study.

A complete history and clinical record including patients age, gender, body mass index (BMI), type of MI, and any existing medical history were recorded for all the patients enrolled in the study. In addition, a physical examination and electrocardiography were done to assess wall motion abnormalities and left ventricular (LV) function. After obtaining informed consent, blood samples were collected to record the blood sugar levels i.e., fasting blood sugar (FBS) and postprandial blood sugar (PPBS) using Trinder’s glucose oxidase method which read at 505/670 nm [16]. Also, thyroid profiles, including TSH (using an ultrasensitive sandwich chemiluminescent immunoassay) and fT4 and fT3 (using a competitive chemiluminescent immunoassay) were recorded. Mortality outcomes were evaluated with respect to thyroid hormone profiles.

After proper coding, the data was analyzed using SPSS version 26.0 (IBM Corp., Armonk, NY, USA). To check the normality of the data, the Kolmogorov-Smirnov test was used where normal distribution was observed. Descriptive analyses, including frequency, percentage, mean, SD, and range, were used to present the data. For categorical data, chi-square was used while independent t-tests and ANOVA were used for quantitative data. Statistical significance was determined at a 5% significance level with a 95% confidence level (p<0.05).

## Results

In total, 100 participants, consisting of 35 males (35%) and 65 females (65%), were included in this study. In terms of age distribution, 22 patients were classified as being above 60 years old, 62 fell within the age range of 40-60 years, and 16 among aged between 18-40 years. Most of the patients ranged from 28-87 years old. Medical history revealed 67 patients as known hypertensives and 62 patients were known diabetics. Nevertheless, none of the individuals presented a medical background of heart or thyroid issues. When assessed by BMI, 13 patients were found obese (BMI >27.5), 45 patients were overweight (BMI 23-27.4), 39 patients were normal (BMI 18.5-22.9), and three were underweight (BMI <18.5). Among all patients diagnosed with ACS, it was observed that 39 patients were classified STEMI group, 37 patients under the NSTEMI group, and 24 patients with UA respectively (Table 1).

**Table 1:** Distribution of demographics and clinical variables among patients with acute coronary syndrome (ACS) %=percentage, n=number of participants, BMI=Body Mass Index, STEMI=ST-elevated myocardial infarction, NSTEMI=non-ST-elevated myocardial infarction, UA=unstable angina

Variables	n	%
Age (years)		
18-39	16	16
40-59	62	62
≥60	22	22
Gender		
Male	35	35
Female	65	65
Medical History		
Diabetes	62	62
Hypertension	67	67
BMI		
<18.5	3	3
18.5-22.9	39	39
23- 27.4	45	45
>27.5	13	13
Types Of MI		
NSTEMI	37	37
STEMI	39	39
UA	24	24

**Figure 1:**
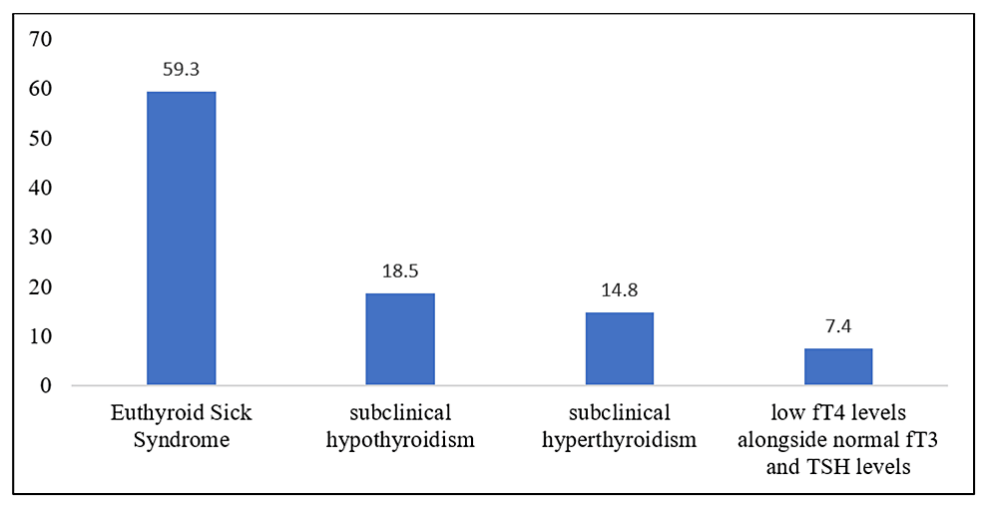
Distribution of types of thyroid dysfunction fT4=free thyroxine, fT3=free triiodothyronine, TSH=thyroid stimulating hormone

Among the cohort of 27 individuals exhibiting abnormal thyroid hormone profiles, it was observed that 16 patients (59.3%) presented with euthyroid sick syndrome, five patients (18.5%) displayed subclinical hypothyroidism, four patients (14.8%) exhibited subclinical hyperthyroidism, and two patients (7.4%) manifested low fT4 levels with normal fT3 and TSH levels. The findings are depicted in Figure 1.

Table 2 shows the association of demographic and clinic variables with the thyroid hormone profile of the patients with ACS. It was observed that 31.25% (5/16) patients with an abnormal thyroid profile were less than 40 years of age, 27.41% (17/62) patients were between 40 and 60 years of age, and 22.72% (5/22) were more than 60 years of age. These differences were found to be statistically significant (p=0.002). Regarding gender distribution, 31% (11/35) of patients with an abnormal thyroid profile were males, while 25% (16/65) were females. The gender distribution exhibited insignificant statistical variation between the groups (p=0.10). However, a notable distinction emerged when BMI was recorded, where 61.53% of patients with abnormal thyroid function were obese (BMI >27.5), 24.44% were overweight (BMI 23-27.4) and 17.94% of patients were normal (BMI 18.5-22.9), and this difference was found to be statistically significant (p=0.002). Concerning medical history, 30.64% of diabetic patients and 23.8% of hypertensive patients had an abnormal thyroid profile. However, the difference was found to be statistically insignificant (p=0.23).

**Table 2:** Association of demographics and clinical variables with the thyroid profile of patients with acute coronary syndrome (ACS) p<0.05=significant, df=degree of freedom, MI=myocardial infarction, STEMI=ST-elevated myocardial infarction, NSTEMI=non-ST-elevated myocardial infarction, UA=unstable angina

Variables	Patients with thyroid dysfunction n (%)	Patients with normal thyroid profile n (%)	Total patients n (%)	Chi-square test value	df	p value
Age						
<= 40	5 (31.25)	11(68.7)	16	63.10	2	0.002
41-60	17 (27.4)	45 (72.5)	62			
>60	5 (22.7)	17 (77.2)	22			
Gender						
Male	11 (31.5)	24 (68.5	35	55.09	1	0.102
Female	16 (25.)	49 (75)	65			
BMI						
< 18.5	1 (33.3)	2 (66.7)	3	14.5	3	0.002
18.5-22.9	7 (17.9)	32 (82.05)	39			
23- 27.4	11 (24.4)	34 (75.5)	45			
>27.5	8 (61.5)	5 (38.4)	13			
Medical History						
Diabetic	19 (30.6)	43 (69.4)	62 (62)	58.2	1	0.23
Hypertension	16 (23.8)	51 (76.2)	67(67)			
Type of MI diagnosed						
NSTEMI	8 (21.6)	22 (78.4)	37 (37)	3.101	2	0.54
STEMI	13 (28.2)	26 (66.6)	39 (39)			
UA	5 (20.3)	19 (79.1)	24 (24)			

When different types of MI were assessed with thyroid profiles, it was observed that 28.2% (13/39) patients in the STEMI group exhibited abnormal thyroid hormone profiles, followed by 21.6% (8/37) in the NSTEMI group, and 20.3% (5/24) among UA group. However, the difference was found to be statistically insignificant (p=0.54).

Table 3 shows the correlation of thyroid profile with health outcomes among abnormal thyroid function patients with ACS. In the present study, a total of nine patients (9%) died with ACS. The mean value for the fT3 (normal range 1.5 to 4.1 pg/ml) was 2.37 pg/ml for the patients who improved and 1.61 pg/ml for expired patients, and the difference was found to be statistically significant (p=0.002). Whereas the mean value for fT4 (normal range = 0.8 to 1.90 ng/dl) for both improved and expired patients was 1.12 ng/dl, and the difference was found to be statistically significant (p=0.009). And for TSH (normal range = 0.42 to 5 mIU/ml) the mean value for improved patients was 2.58 uIU/ml and 2.37 uIU/ml for expired patients, and the difference found was statistically significant (p=0.006). The mean values for rT3 among expired and improved patients were found to be 0.6 and 2.48 and this difference was found to be highly statistically significant (p=0.001).

**Table 3:** Association of thyroid hormone profile with health outcome of the patients suffering from acute coronary syndrome (ACS). p<0.05=significant, TSH=thyroid stimulating hormone, fT4=free thyroxine, fT3=free triiodothyronine, rT3=reverse triiodothyronine

	Group	n	Mean ± Standard Deviation				p-value
fT3	Improved	93	2.37 ± 0.008				0.002
	Expired	7	1.61 ± 0.007				
fT4	Improved	93	1.12 ± 0.0024				0.009
	Expired	7	1.12 ± 0.0029				
TSH	Improved	93	2.58 ± 1.01				0.006
	Expired	7	2.37 ± 1.51				
rT3	Improved	93	0.60 ± 1.2				0.001
	Expired	7	2.48 ± 1.01				

## Discussion

ACS has emerged as a serious condition that warrants severe attention due to its substantial impact on the homeostasis of the thyroid gland, resulting in consequences related to morbidity and mortality [17]. The impact of thyroid hormones on conditions such as heart failure [9,18], systemic arterial hypertension [5], atherosclerosis [13], dyslipidemia, and cardiopulmonary operations has been extensively examined in varied research [19,20]. This cross-sectional study endeavors to appraise the significance of thyroid-releasing hormone among patients suffering from ACS and emphasize its relevance in predicting major adverse cardiovascular events (MACE).

The demographic characteristics of the sample were carefully recorded to ensure a characteristic representation of the ACS population. The study findings showed female preponderance (65%), with the majority of patients (62%) belonging to 40-60 years of age, having a history of comorbidities including diabetes (62%), hypertension (67%), and being overweight (45%). The majority of the patients (39%) were diagnosed with STEMI. The above findings were found in accordance with the study conducted by Jabbar et al. [15] and Abdulaziz Qari [5]. Conversely, male predominance among age groups older than 60 years was reported by Sah et al. [8]. Discrepancies among these findings may be attributed to the different study populations and settings.

The major findings of the present study showed that 27% of patients have abnormal thyroid hormone profiles. However, a lower prevalence of abnormal thyroid hormones among patients with ACS was reported by Sah et al. (25.0%) [8], Abdulaziz Qari (23.3%) [5], and Bayrak et al. [6], whereas a higher prevalence was reported by Mathur et al. (31%) [21], and Adawiyah et al. (53%) [22]. These differences might be due to the varied inclusion criteria in different studies. Within the scope of our investigation, it was determined that 59.3% of patients with ACS were diagnosed with euthyroid sick syndrome, 18.5% of participants exhibited subclinical hypothyroidism, 14.8% displayed subclinical hyperthyroidism, and 7.4% manifested low fT4 levels alongside normal fT3 and TSH levels. These results were aligned with the findings reported by Rahman et al. [23]. This corroborates the fact that heart disorders such as ACS have been linked with an alteration in the level of serum thyroid hormone levels [24,25].

Our study compared the general characteristics with the thyroid profile of the patients with ACS. It was noted that a significant portion of the patients (31.2%) with ACS who had abnormal thyroid hormone profiles were aged less than 40 years, while the lowest prevalence of abnormal thyroid profiles was seen among patients above 60 years of age. These results were aligned with the results reported by Sengottaiyan et al. [25]. This finding can be substantiated by existing evidence, which reported that in very old populations, a definite degree of downregulation mechanism of thyroid hormones existed, which might represent a protective factor and make them less vulnerable to abnormal thyroid profiles [26]. Regarding gender distribution, though statistically insignificant, males (31.42%) were found to have a higher prevalence of abnormal thyroid hormone levels than females (24.61%). These findings were found to be similar to the results reported by Sengottaiyan et al. [25] and Sah et al. [8], suggesting that the occurrence of socioeconomic status (SES) was not influenced by sex. Thyroid hormones have a well-established and important implication in the regulation of body weight and thus with the incidence of obesity. A similar finding was found in the present study, where a significant portion of the patients were found obese (61%), aligning with the results reported by Sengottaiyan et al. [25]. Hence, BMI is recommended as a strong predictor of thyroid dysfunction in patients with ACS.

In the current study, a greater incidence of aberrant thyroid hormone profiles was observed in patients in the STEMI group as compared to the NSTEMI/UA group; however, the difference was found to be statistically insignificant. These results were in accordance with the findings reported by Sah et al. [8], Tuzun et al. [24], and Mathur et al. [21].

Atypical thyroid status is defined as a condition with altered thyroid levels and is categorised into overt hypothyroidism (TSH concentration >4.50 mU/L), subclinical hypothyroidism (TSH concentration >4.50 and <20 mU/L), and overt thyrotoxicosis (TSH concentration <0.10 mU/L). In our study, nine patients died of ACS, and all of them had statistically significant low fT3 and TSH values while having higher rT3 values (p<0.05). Comparable results were reported by Abdulaziz Qari [5], Sengottaiyan et al. [25], and Cerillo et al. [27]. This can be ascribed to the fact that T3 and TSH have a significant influence on gene expression and cell signaling for myocardial contractility. A slight alteration in thyroid hormone status has a profound effect on heart rate and rhythm and hence raises the risk of ACS and related mortality [17,24,28,29].

Our study contributes valuable insights into the importance of thyroid hormone levels in maintaining cardiovascular homeostasis. The above findings not only support the existing literature but also report various predictors for abnormal thyroid hormone in patients with ACS. Our data indicate that thyroid function can be contemplated as a prognostic as well as predictive marker in patients with CVS disorders.

Limitations

Though the study's cross-sectional design facilitates the methodical gathering of all relevant data, this study still has certain limitations, such as a comparatively smaller sample size, a single-center study setting, and shorter follow-ups of the patient. Additionally, we did not make any adjustments for confounding factors such as age, gender, etc.

Hence, we recommend that to obtain comparable results, more multicentric, larger research with standardized inclusion criteria needs to be conducted. In order to assure the validity and reliability of the findings, future studies should use standardized methodology and data collection approaches. Moreover, investigating long-term results and carrying out follow-up evaluations would offer a more thorough grasp of the elements impacting the reported results. Standardized approaches and outcome metrics would also improve the validity and dependability of the results.

## Conclusions

An atypical thyroid status has been found to elevate the likelihood of developing coronary artery disease and experiencing cardiovascular mortality. This condition can impact ventricular function, serum cholesterol levels, as well as heart rate and rhythm. This study reported alterations in the thyroid hormone levels in 27% of patients with ACS. The prevalence of euthyroid sick syndrome was found to be 59.3%, while subclinical hypothyroidism and subclinical hyperthyroidism were reported among 18.5% and 14.8% of patients respectively. There was no gender disparity seen among ACS patients. The study observed a greater occurrence of aberrant thyroid hormone profiles in those aged 40-60 years. The STEMI group had a statistically significant greater prevalence of an aberrant thyroid hormone profile compared to the NSTEMI/UA group (p=0.02). Low fT3, TSH, and high rT3 were found to be important markers of mortality in patients suffering from ACS.
